# Douglas-Fir Seedlings Exhibit Metabolic Responses to Increased Temperature and Atmospheric Drought

**DOI:** 10.1371/journal.pone.0114165

**Published:** 2014-12-01

**Authors:** Kirstin Jansen, Baoguo Du, Zachary Kayler, Rolf Siegwolf, Ingo Ensminger, Heinz Rennenberg, Bernd Kammerer, Carsten Jaeger, Marcus Schaub, Jürgen Kreuzwieser, Arthur Gessler

**Affiliations:** 1 Institute for Landscape Biogeochemistry, Leibniz Centre for Agricultural Landscape Research (ZALF), Müncheberg, Germany; 2 Institute for Forest Botany and Tree Physiology, Albert-Ludwigs University Freiburg, Freiburg, Germany; 3 Sichuan Province Key Laboratory of Ecological Security and Protection, Mianyang Normal University, Mianyang, China; 4 Laboratory of Atmospheric Chemistry, Stable Isotopes and Ecosystem Fluxes, Paul Scherrer Institute (PSI), Villigen, Switzerland; 5 Department of Biology, University of Toronto, Mississauga, Ontario, Canada; 6 Core Facility Metabolomics, Center for Biological Systems Analysis (ZBSA), Albert-Ludwigs University Freiburg, Freiburg, Germany; 7 Swiss Federal Institute for Forest, Snow and Landscape Research (WSL), Birmensdorf, Switzerland; 8 Berlin-Brandenburg Institute of Advanced Biodiversity Research (BBIB), Berlin, Germany; Henan Agricultural Univerisity, China

## Abstract

In the future, periods of strongly increased temperature in concert with drought (heat waves) will have potentially detrimental effects on trees and forests in Central Europe. Norway spruce might be at risk in the future climate of Central Europe. However, Douglas-fir is often discussed as an alternative for the drought and heat sensitive Norway spruce, because some provenances are considered to be well adapted to drier and warmer conditions. In this study, we identified the physiological and growth responses of seedlings from two different Douglas-fir provenances to increased temperature and atmospheric drought during a period of 92 days. We analysed (i) plant biomass, (ii) carbon stable isotope composition as an indicator for time integrated intrinsic water use efficiency, (iii) apparent respiratory carbon isotope fractionation as well as (iv) the profile of polar low molecular metabolites. Plant biomass was only slightly affected by increased temperatures and atmospheric drought but the more negative apparent respiratory fractionation indicated a temperature-dependent decrease in the commitment of substrate to the tricarboxylic acid cycle. The metabolite profile revealed that the simulated heat wave induced a switch in stress protecting compounds from proline to polyols. We conclude that metabolic acclimation successfully contributes to maintain functioning and physiological activity in seedlings of both Douglas-fir provenances under conditions that are expected during heat waves (i.e. elevated temperatures and atmospheric drought). Douglas-fir might be a potentially important tree species for forestry in Central Europe under changing climatic conditions.

## Introduction

Global climate change is expected to affect forest growth and productivity [1], [2]. The projected increase in air temperature is the most direct and best predictable effect of increasing tropospheric CO_2_ concentrations [3]. In addition to a continuous increase in air temperature, the frequency of climate extremes, including heat waves, has been predicted to strongly increase [2], [4]–[7]. Tree growth and physiological performance will depend on both the acclimation to increased temperatures and the resistance against extreme events such as the hot-dry summer 2003 in Central Europe [8]. Our insights into the adaptation and acclimation potential of tree species towards climatic change are still incomplete and especially the effects of heat waves i.e. periods of increased temperature in concert with drought [8]–[10] on the physiology of trees are not well understood, e.g., [11].

Compared to deciduous tree species, evergreen species show a weak growth increase [12] or no growth response to constant, yet, moderate temperature increases [13]. In Douglas-fir, the 2003 drought and heat wave showed diverging effects on radial growth ranging from no response at moist sites to significant decreases at dry sites in Germany and Switzerland [14], [15]. A species' response to increased temperature depends on its thermal optimum for photosynthesis and growth, and its current operation either at or below the optimum [12]. Rising temperatures may stimulate assimilation, physiological performance, and growth below the thermal optimum, but may lead to reduced performance above species- or provenance-specific thresholds [16], [17].

Changes in temperature might not only affect photosynthesis and thus carbon supply for growth but also sink activity [18] and, as an underlying mechanism, the allocation of assimilated C to different metabolic pathways [19]. Shifts in the commitment of metabolic pathways can be detected by changes in the apparent respiratory carbon isotope fractionation [20]. Leaf respired CO_2_ is generally ^13^C-enriched compared to the putative respiratory substrate [20]–[22], mainly due to fragmentation of the substrate molecule (fragmentation fractionation) [23], [24] and potentially due to enzyme related isotope effects [20], [25]. During glycolysis, the C-1 of pyruvate derived from ^13^C-enriched C-3 and C-4 of glucose is decarboxylated by pyruvate dehydrogenase (PDH) and, consequently, the PDH reaction releases ^13^C-enriched CO_2_ [23]. The remaining part of the pyruvate molecule can enter the tricarboxylic acid (TCA) cycle during which relatively depleted CO_2_ is released. Alternatively, the remaining part of pyruvate might be used for the production of acetogenic lipids and various secondary metabolites cf. [20]. Assuming that the whole pyruvate molecule were oxidised in the TCA cycle, the δ^13^C of CO_2_ released from respiration would equal that of the glucose substrate. The more pyruvate that is committed to metabolic pathways other than the TCA cycle, the higher the contribution of the PDH-released ^13^C enriched CO_2_ to the total respiratory CO_2_ emission, and the more negative the apparent respiratory fractionation.

Moreover, metabolite profiling provides information about the accumulation of precursors of secondary metabolites or stress related compounds [19]. For instance, proline accumulation is associated with various environmental stresses see, e.g., [26], [27], but can also be toxic to cells [28]–[31], especially under heat stress [32]. Alternative osmolytes such as polyols [33], [34] might be crucial for maintaining leaf turgor under conditions of drought and high temperatures. Especially the polyol D-pinitol is known to be present in large amounts in conifers e.g. [35] and might play an important role in osmotic adjustment in these plants. Moreover, heat stress is known to generally upregulate metabolites related to the shikimate pathway [19], which is related to the synthesis of cell wall components, lignin, aromatic amino acids, phenylpropanoids and flavonoids, and is also closely linked with carbohydrate metabolism [36].

In Central Europe, Douglas-fir (*Pseudotsuga menziesii* (Mirb.) Franco) is often discussed as a potential alternative for the economically important Norway spruce (*Picea abies* (L.) Karst.), the performance of which could be seriously threatened by climate change [37]. Douglas-fir is a remarkably productive species with a high economic potential and it is assumed to display a comparably high resistance against drought and heat [38], [39]. Within an extended natural range, Douglas-fir populations grow under various climatic conditions and these different environmental drivers may act as selective force on the genetic and physiological growth response of different provenances [40], [41]. For instance, our current understanding of water relations in Douglas-fir seedlings suggests that populations from regions with relatively cool winters and arid summers are potentially best adapted to warm and dry conditions [42]. With climate change, future summer temperatures in Central Europe will probably more frequently exceed the thermal optimum for photosynthesis in Douglas-fir (10–20°C) [43], [44]. Heat waves will be most likely accompanied by reduced precipitation and high water vapour pressure deficit (VPD) resulting in atmospheric drought [7]. This calls for a better understanding of the interactive impact of heat and atmospheric drought on Douglas-fir physiology and growth in order to inform forest managers about the potential of this species. One issue that is of major importance is the impact of heat and drought on tree seedlings as they represent the future of a forest stand and there are indications that the acclimation to environmental stresses strongly depends on ontogeny [45] and that seedlings are more intensively affected by environmental stressors compared to adult individuals [46].

The aim of this study was to identify seedling physiological and growth responses of Douglas-fir from two different provenances to a simulated heat wave accompanied by atmospheric drought. We examined the two provenances Monte Creek (MC) and Pend Oreille (PO), originating from the *menziesii*-*glauca* transition zone in southern interior British Columbia and the *glauca* zone in Northwest Washington State, respectively (Table 1) [cf. 47–50]. These provenances are originating from the rather dry part of the natural range of Douglas-fir, in comparison to the coastal areas of Washington and British Columbia. The summer air temperatures are comparable for the two provenances, however, the MC provenance region receives only approx. half the amount of rainfall compared to the PO region and thus individuals from the MC provenance might be better adapted to heat accompanied by atmospheric drought.

**Table 1:** Provenances, geographic location and climatic conditions in the regions of origin.

Name	Variety	Elevation	Latitude	Longitude	MAT	MAP	MST	MSP	CMD
		[m asl]	N	W	[°C]	[mm]	[°C]	[mm]	[mm]
***Canada, Southern Interior British Columbia, Thompson Okanogan***									
Monte Creek	interior/transition	850±50	50.7	−120	5.2	362.0	13.56	171.0	433.0
***USA, Northeast Washington State, Okanogan Highlands***									
Pend Oreille	interior	925±75	48.3±0.7	−117.7±0.7	6.5	735.7	14.78	219.5	396.7

Climatic parameters as mean annual temperature (MAT), mean annual precipitation (MAP), mean summer (May-Sep) temperature (MST) and precipitation (MSP), Hargreaves climatic moisture deficit (CMD, sum of the monthly difference between a reference evaporation and precipitation) is calculated with Climate WNA as described in Wang et al. (2012).

To examine effects of heat waves on physiology and growth of Douglas-fir, seedlings were grown under high temperature (control +10°C) combined with high vapour pressure deficit (control +1.6 kPa) as well as under control conditions (20°C and 0.35 kPa) for more than three months (Table S1). The 2003 heat wave showed whole July temperature anomalies (compared to 2001) of up to +10°C for large areas of Central Europe [51] indicating the realistic temperature range of our treatment. We analysed plant biomass, carbon stable isotope composition as an indicator for time integrated intrinsic water use efficiency (IWUE  =  assimilation/stomatal conductance) [52], apparent respiratory fractionation in order to assess temperature-dependent changes in the commitment of substrate to the TCA cycle [25], [53], as well as the profile of polar low molecular metabolites.

We hypothesize that (1) the increased temperature together with atmospheric drought (i.e. elevated VPD) will reveal metabolic plasticity where precursors are fed into alternative metabolic pathways resulting in altered metabolite profiles. We further assume (2) that heat and atmospheric drought during three months lead to reduced biomass production and changes in biomass allocation patterns. Finally we hypothesize (3) that the MC provenance is less affected by the combined heat and atmospheric drought impact as a consequence of its potential adaptation to the lower water availability at the area of origin.

## Materials and Methods

### Plant material and growing conditions

#### Ethics statement

We did not perform field work but worked with seedlings under controlled conditions in climate chambers. The study did not involve endangered or protected species. Douglas-fir seedlings were obtained from forest tree nurseries (Nursery Services Interior, Vernon, Canada and Webster Forest Nursery, Olympia, WS, USA) and no specific permission was required for buying the seedlings. Import to Germany and growing plants under quarantine regulations was permitted by the Regierungspräsidium Freiburg; Pflanzengesundheitsdienst, Freiburg, Germany.

Three to four years old seedlings of the Douglas-fir provenances Monte Creek (MC) and Pend Oreille (PO) (see Table 1) were grown in environmental chambers in 4 L pots with substrate consisting of commercial potting soil (1 part Anzucht- und Pikiererde, Ökohum, Herbertingen, Germany; 1 part perlite; 1 part sand). The substrate was supplied with long-term fertilizer (Osmocote Exact high-end 5–6, 15N+9P+12K+2Mg, 3 g L^−1^ substrate, The Scotts Company, LLC, Marysville, OH, USA/ICL, Tel Aviv, Israel). The chambers were illuminated by sodium-vapour lamps (NC 1000-00/-01/-62, Narva, Plauen, Germany, with 6, 8 and 10–11% red light, respectively).

Over a period of 78 days, plants were stepwise acclimatized to control conditions (Table S1). The following treatment phase covered 92 days and started after full leaf development. The aim of this approach was to simulate a heat wave comparable to the one in Europe in 2003, which occurred in mid summer after foliage had been fully developed. During the first 10 days of acclimation, the temperature was raised from 10/10°C (light/dark period) to 20/15°C. Plants in the treatment group were exposed to 25/20°C for three days before the 30/25°C treatment started. Coniferous needle leaves are strongly coupled to the environment [54], resulting in leaf temperature equalling air temperature. During the acclimation period, the photoperiod was extended from 12 h to 16 h and the light intensity was raised from 250 to 600 µmol m^−2^ s^−1^ PPFD to be above the light saturation point of Douglas-fir [55].

During the treatment phase, VPD was held at 0.35 kPa (in the light period) in the control and 1.91 kPa in the treatment, the latter assumed to represent atmospheric drought. In both groups, soil water content was kept at optimum levels (approx. at field capacity). During the time of experimentation, the plants had three needle age classes developed at the beginning of the current year 2010 (N10), in the previous year (N09), and in the year 2008 (N08).

Climate data for the region of origin from the two provenances examined was obtained with the software ClimateWNA by downscaling PRISM monthly data [56] for the reference period (1961–1990), and calculating seasonal and annual climate variables for the specific locations based on latitude, longitude, and elevation (Table 1) as described in [57]. For the MC provenance, the geographic location as provided by the seedling supplier (Nursery Services Interior, Vernon, BC, Canada) was used as input parameter. The PO provenance originates from the seed zone Pend Oreille described by [49]. A grid of geographic locations within the seed zone was used as input for ClimateWNA and the resulting multiple climate data were averaged. Annual precipitation sum (mm), mean summer precipitation sum (mm) and mean annual temperature (°C) in the PO zone of origin are around 374 mm, 48 mm and is 1.26°C higher than in the MC zone of origin (Table 1).

### Experimental design and biomass sampling

We conducted repetitive sampling during a period of 92 days after treatment start (DAT), including measurements during a diel course and a complete harvest of the plants at the end of the experimental period. On DAT 1 and 41 we sampled current-year needles of 20 plants (N = 5) for carbon isotope analysis. We conducted a full diel course on DAT 64 with 12 plants (N = 3) and six sampling time points (four during the light and two during the dark period) to study the carbon isotopic composition of respired CO_2_ and of potential organic sources for respiration, as well as respiration rates. At each time point (2, 6, 10, 14, 18, and 22 h±2 h), we sampled current-year needles, fine roots, and respired CO_2_ from canopy chambers (see below). Needle and root samples were taken from separate plants to avoid interferences with the CO_2_ samples. At the end of the experimental period, from day 76 to 92 after treatment start, 36 plants were completely harvested for biomass, isotope and metabolite analyses, and separated into needles (subdivided into 3 age classes), twigs, stems (subdivided into bark and wood), fine and coarse roots, and measured for fresh weight. Subsamples were dried at 105°C for 24 h for assessing dry weight while the rest of the samples was immediately frozen in liquid nitrogen and stored in −80°C until further analyses.

### Stable isotope analyses of organic matter and CO_2_

δ^13^C in organic matter was determined in the water soluble fraction (WSOM; water soluble organic matter) of needles according to [58], [59]. 250 µl of the extracts were dried under vacuum and stored in a desiccator. The isotopic composition of WSOM is an excellent proxy for sugars and thus recent assimilates [58]. In addition, δ^13^C was determined in the total organic matter fraction (bulk) of several plant compartments, i.e. in the dried and homogenised plant material.

For δ^13^C analysis, approx. 0.3 mg of the dried homogenised total organic matter samples and the dried WSOM extracts samples were combusted in an Flash HT elemental analyzer (ThermoFinnigan, Bremen, Germany) coupled via a Conflo III interface to an isotope ratio mass spectrometer (Delta V Advantage, ThermoFisher, Bremen, Germany). Carbon isotopic values were expressed in δ notation relative to the Vienna Pee Dee Belemnite (VPDB). The precision for measurements as determined by repeated measurements of standards (n = 10) was better than 0.10 ‰.

For the assessment of δ^13^C in canopy respired CO_2_ (δ^13^C_R_), we installed Perspex respiration chambers around the upper part of the canopy (5.39 L volume, custom-made production, Herbert Geißler GmbH & Co. KG, Freudenberg, Germany) according to [60]. The chambers enclosed the needles and twigs from the approx. upper half of the crown and were supplied with interior fans to ensure mixing of air, sealed with an inert plastic material (Terostat VII, Henkel Teroson GmbH, Heidelberg, Germany), completely darkened with aluminium tape to prevent photosynthesis, and provided with air-tight septa to allow gas sampling with hypodermic needles and syringes. The gas samples were taken 0, 1, 5, 12 and 20 min after closing the chambers and subsequently transferred into pre-evacuated exetainers (12 ml, Labco Ltd, High Wycombe, UK). The chambers were removed directly after each sampling phase.

Gas samples were analysed for δ^13^C and [CO_2_] in an isotope ratio mass spectrometer (Delta V Advantage, ThermoFisher, Bremen, Germany) coupled to a gas bench as described by [61]. δ^13^C_R_ was determined with mixing models using the Keeling plot approach [62], [63] and the Miller-Tans approach [64]. The [CO_2_] range can impact the outcome of mixing models [65]. In our case, both approaches resulted in very similar results and we thus applied an average for δ^13^C_R_. Respiration rates were calculated from the linear increase in CO2 concentration over time and based on the total leaf, twig, and stem area. Apparent respiratory fractionation (aΔ_R_) was calculated as the difference between δ^13^C of current-year needle WSOM and δ^13^C of respired CO_2_ according to [58].

### Analysis of metabolites by gas chromatography–mass spectrometry (GC-MS)

Polar low-molecular-weight metabolites were extracted from N09 and N10 needles and derivatised according to a modified method from [66] and [67]. For each sample, approximately 50 mg of homogenized frozen tissue powder was weighed into a pre-frozen 2 mL round-bottom Eppendorf tube and 500 µL of cold 85% (v/v) methanol (Sigma) were added as extraction medium. 1 µg ml^−1^ ribitol was used as internal standard. Tubes were rapidly heated to 65°C and shaken at 1,400 RPM for 15 min after brief vortex. 50 µL aliquots of supernatant were dried under vacuum in 1.5 ml microfuge tubes after centrifugation. Dried extracts were methoximated by adding 20 µL of a 20 mg mL^−1^ solution of methoxyamine hydrochloride in anhydrous pyridine (Sigma) and incubated at 30°C for 90 min with shaking at 1400 RPM. For trimethylsilylation, 70 µL of N-methyl-N- (trimethylsilyl) trifluoroacetamide (MSTFA; Sigma) was transferred to each tube and incubated at 37°C for 30 min with 1,400 RPM shaking. 10 µL of an n-alkane retention index calibration mixture [n-Alkane- Mix 16 (C10-C40 even), Cat.-No.:14640, concentration: 50 µg ml^−1^ in n-hexane; Neochema, Germany] was then added to each sample. After short vortex, reaction mixtures were centrifuged at 14,000 g, 20°C for 2 min and then 80 µL of supernatant were transferred to amber GC-MS vials with low volume inserts and screw top seals (Agilent Technologies, Palo Alto, CA, USA) for GC-MS analysis.

Derivatised metabolite samples were analysed on an Agilent GC/MSD system comprised of an Agilent GC 7890A gas chromatograph (Agilent Technologies, Palo Alto, CA, USA) fitted with a GERSTEL MultiPurpose Sampler (MPS2-XL, GERSTEL, Mülheim, Germany) and 5975C Inert XL EI/CI MSD quadrupole MS detector (Agilent Technologies, Palo Alto, CA, USA). The capillary column used was HP-5MS 5% Phenyl Methyl Silox, length: 30 m, diameter: 0.25 mm, film thickness: 0.25 µm (Agilent Technologies, Palo Alto, CA, USA). GC-MS run conditions were set up according to [67] with some slight adaptations. The GC column oven was held at the initial temperature of 80°C for 2 min and then to 325°C at 5°C min^−1^ before being held at 325°C for 10 min. Total run time was 61 min. Transfer line temperature was 280°C. MS source temperature was 230°C.

The raw data files were processed with the free AMDIS (automated mass spectral deconvolution and identification system, version 2.69) software supplied by NIST (National Institute of Standards and Technology, Gaithersburg, MD, USA). Mass spectra were searched against a user-defined metabolite database based on the Golm Metabolome Database [68] and identified based on retention index and spectrum similarity match. A relative quantification of metabolite peaks was done by calculating the areas of the extracted ion chromatograms. The area value for each metabolite was standardised by sample weight, then normalized for the area measured for the internal ribitol standard, and corrected for blank values where only solvents and derivatisation reagents were measured. Metabolites detected in less than 50% of all the replicates of each provenance and needle age class were discarded for the comparison between treatments and controls. Treatment specific differences of the corrected and normalised areas are given as log ratios of treatments *vs* controls.

### Statistical analyses

All measured parameters were characterized by descriptive statistics (means and standard deviations of the means). Statistical analyses were carried out with R 3.0.2 (R Development Core Team 2013). Normality of residuals was tested with the Shapiro-Wilk test and was rejected when p-values were smaller than 0.1. Homogeneity of variances was tested with the Fligner-Killeen test on grouped data and rejected when p-values were smaller than 0.05. A 2-way analysis of variance was conducted to assess the effects of provenance, treatment and their interactions. In case of non-normality of residuals, ANOVA was performed on ranks. In case of interacting effects in the 2-way ANOVA, a 1-way ANOVA was conducted for single parameters in order to determine effects separately. Tukey's post-hoc was used following the ANOVA analysis for multiple comparisons among the groups.

## Results

### Plant biomass

Treatment-specific effects on the dry weight of Douglas-fir seedlings were only observed for needles of the oldest age class N08 (Figure S1A). The biomass of these needles decreased significantly under high temperature and VPD. All other needle age classes and tissues as well as total biomass were not significantly affected by high temperature and atmospheric drought treatment.

There was no significant temperature effect on the ratio between above- and below-ground biomass, indicating that above- to below-ground biomass allocation was not affected by the treatment (Figure S1B). However, the ratio between fine-root and coarse-root biomass was significantly decreased, whereas the ratio between current-year needles and fine roots was increased.

### Carbon isotope composition of plant organic matter

**Figure 1:**
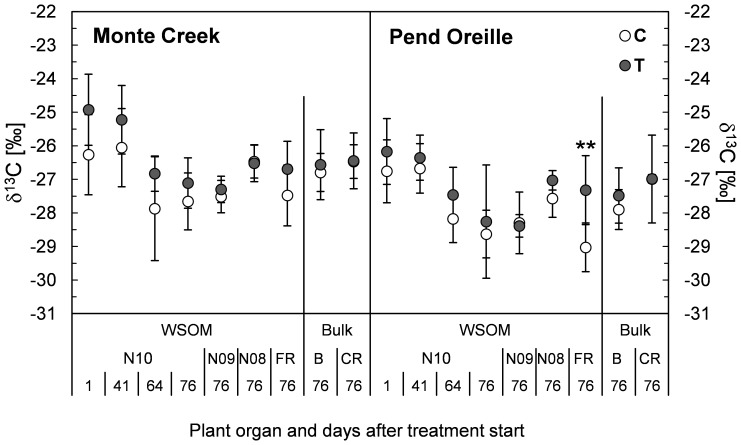
Effects of increased temperature and atmospheric drought on δ^13^C. δ^13^C is shown for water soluble organic matter (WSOM) in current year's needles (N10), older needles (N09, N08) and fine roots (FR), and in bulk material of bark and coarse roots (B, CR) from Douglas-fir seedlings of the provenances Pend Oreille and Monte Creek in control C (20°C, 0.35 kPa, white circles) and temperature/atmospheric drought treatment T (30°C, 1.91 kPa, grey circles). Asterisks indicate significant differences between treatments C and T (Tukey test, *** for p<0.001, ** p<0.01, * p<0.05). Data shown are mean values ± SD (N = 3 (64 DAT) – 10 (76DAT)). Numbers 1, 41, 64, 76 in the x-axis caption indicate the time point of harvest after the onset of the experiment.

In both provenances and in both treatments, δ^13^C in WSOM of current-year needles (N10) decreased during the treatment period by up to 1.9‰ for MC and 2.0‰ for PO (Figure 1). δ^13^C in WSOM of all analysed needle age classes was significantly affected by provenance at the end of the treatment period (p<0.005 for N10) with lower values for PO. The heat and high VPD treatment did not affect δ^13^C of WSOM in the three needle age classes (Figure 1). Significant effects of high temperature and VPD on δ^13^C were found only in fine root WSOM of PO (Figure 1). Bulk total organic matter δ^13^C in coarse roots and bark were also not affected by treatment,

### Respiration rate and apparent respiratory fractionation

**Figure 2:**
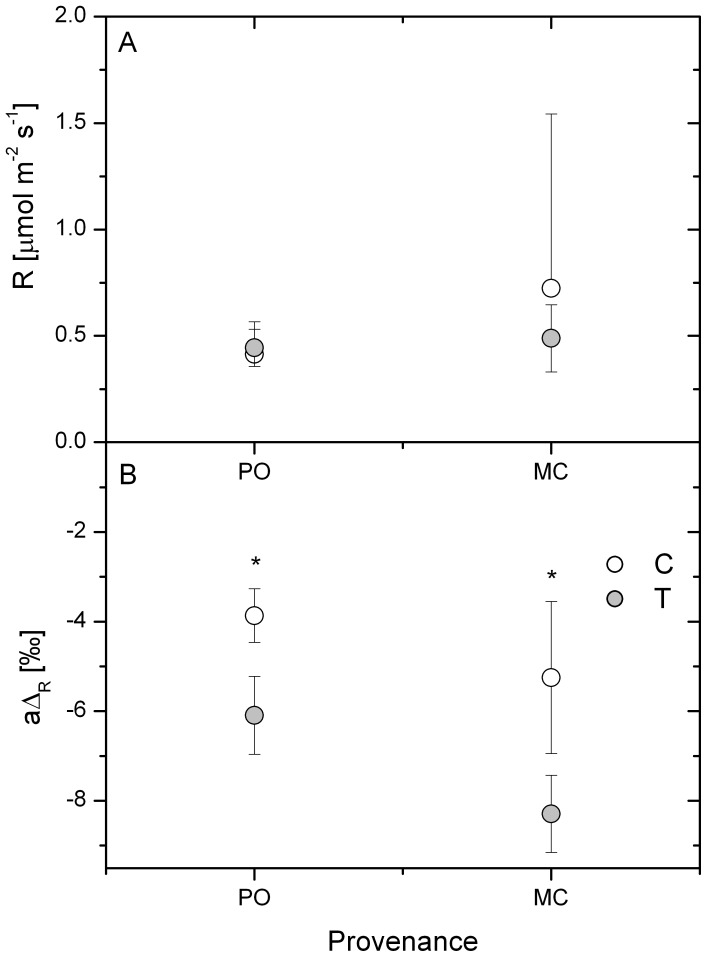
Canopy dark respiration rate (A) and apparent respiratory isotope fractionation (aΔ_R_) (B) in Douglas-fir seedlings of the provenances Pend Oreille (PO) and Monte Creek (MC). The fractionation is based on the difference of δ^13^C measured in water soluble organic matter of needles and δ^13^C in canopy respired CO_2_ as average during a diel course. Data shown are mean values ± SE (N = 3). Significance levels are given for differences between treatments as revealed by Student's t-test (* p<0.05). The controls (20°C, 0.35 kPa) are indicated by white circles and the temperature/atmospheric drought treatment T (30°C, 1.91 kPa) by grey circles.

We determined canopy dark respiration rate and the difference between δ^13^C of WSOM and CO_2_ (apparent respiratory fractionation; aΔ_R_) during a diel course. There was no indication of a significant increase in aΔ_R_ during the light period (data not shown), revealing the absence of light enhanced dark respiration [58], [69]. There was also no significant difference between dark respiration in the light and the dark phase, and therefore we calculated a daily average of the values (Figure 2).

Respiration rates did not differ significantly between treatment and control for both provenances and they did not show provenance-specific differences. In contrast, in both provenances aΔ_R_ decreased significantly with higher temperature and atmospheric drought by 2.2‰ for PO and 3.0‰ for MC. Although there was not a statistically significant difference, we observed a trend towards a lower aΔ_R_ in MC compared to PO independent of the treatment.

### Metabolic profile

**Figure 3:**
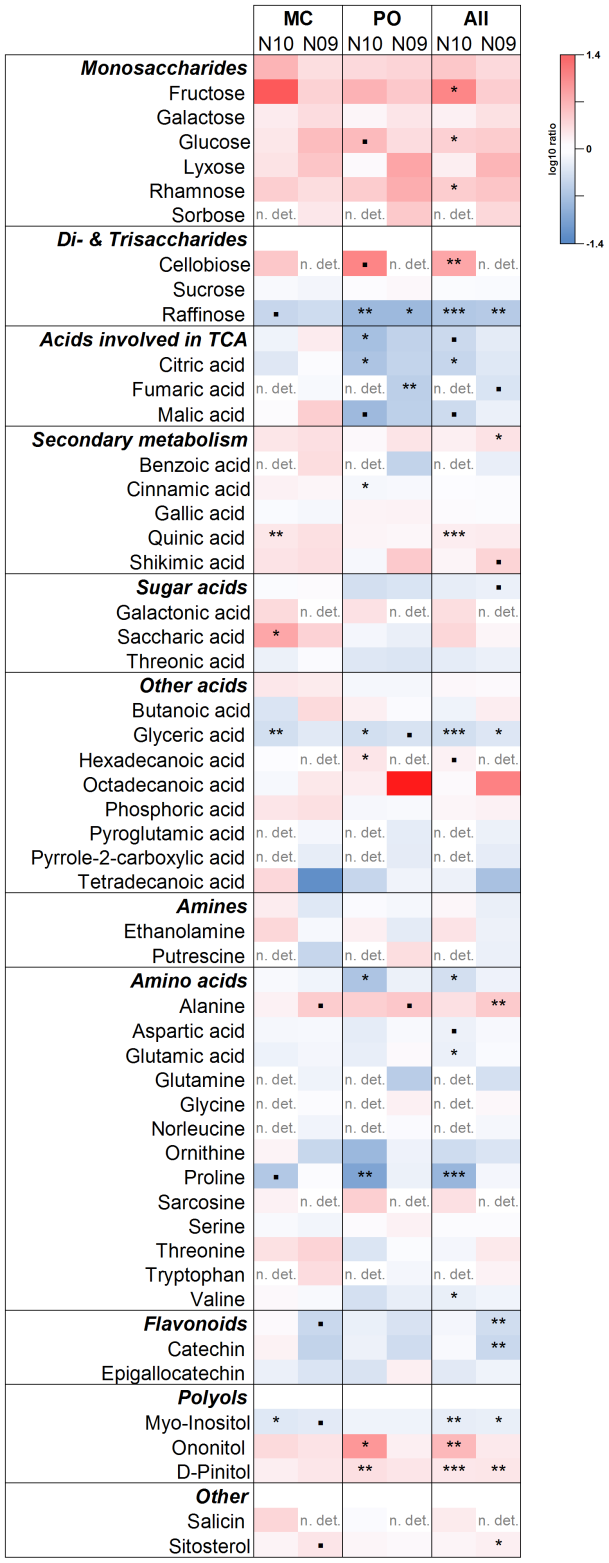
Effects of increased temperature and atmospheric drought on the metabolite profile of two Douglas-fir provenances (Monte Creek and Pend Oreille) and over all provenances (All) in current year's (N10) and last year's needles (N09). Treatment effects are shown as the log10 ratios of temperature/atmospheric drought treatment (T) *vs.* controls (C) obtained from the mean values of the relatively quantified metabolite peaks of the two treatments (N = 4–6 for for each treatment N10 and N = 3–5 for N09). Significance levels are given for group differences (T vs. C) and were obtained by a post-hoc Tukey test (*** for p<0.001, ** p<0.01, * p<0.05,. p<0.1). n. det.: the compound was not detected in at least 50% of the replicates of each provenance and needle age class.

We analysed changes in the metabolite profiles due to the treatment in current-year (N10) and previous-year needles (N09) for the two provenances separately, and averaged over both provenances (Figure 3). In both provenances and both needle age classes, there was a general trend for an increase in monosaccharide concentrations in the high temperature treatment. The difference was significant for glucose in N10 needles of PO and for fructose, glucose and rhamnose in N10 needles averaged over both provenances. While sucrose concentrations did not change, raffinose decreased in both needle age classes (average of both provenances). Cellobiose increased in elevated temperature exposed plants in N10 in both provenances (significant in PO) and as a consequence the average for the two provenances increased significantly. Within the group of polyols, myo-inositol decreased for the average of the two provenances in both age classes, but when analysed separately for each provenance, the decrease was only significant for MC. As a provenance average, D-pinitol and ononitol concentrations increased in both needle age classes and N10 needles, respectively, as a response to the treatment. These increases were most pronounced and significant in the provenance PO.

Organic acids involved in the TCA cycle were decreased upon the temperature and atmospheric drought treatment for the average of the two provenances: citrate and malate decreased in N10 and fumarate in N09 needles. This decrease was more prominent in the provenance PO and there was no significant difference between control and treatment in MC.

There was a trend for an increase in precursors and metabolites of secondary metabolite pathways as a consequence of the temperature treatment. This increase was significant for shikimate (N09, average of provenances; p<0.1), cinnamic acid (N10, PO; p<0.05), and quinic acid (N10, PO; p<0.01, and average of provenances; p<0.001).

For sugar acids, we observed an increase in saccharic acid under increased temperature and atmospheric drought (N10, MC), but a decrease in glyceric acid in both provenances (N10, MC, PO and average; N09, PO and average).

Total amino acid concentration decreased significantly in current-year needles (N10) in PO and as average of both provenances. The clearest decrease was observed for proline in N10 of both provenances whereas alanine increased in N09.

Flavonoids and especially catechin decreased significantly (p<0.01) and sitosterol concentrations increased in N09 needles (p<0.01) for the average of the two provenances.

## Discussion

### Plant biomass and δ^13^C were only slightly affected by high temperature and atmospheric drought

In our study, the high temperature and atmospheric drought treatment (Table S1) did not affect total biomass in the two Douglas-fir provenances examined (Figure S1). We designed the experiment so that the new flush of needles was already fully developed by treatment initiation. Cambial activity is assumed to continue after leaf expansion, so that stem and root growth might be affected by the high temperature and atmospheric drought treatment. As expected, current-year needles biomass (N10) was not affected; the same was true for the previous-year needles (N09). The reduction in N08 needle biomass might be explained by needle loss during the experimental period and/or by withdrawal of non-structural compounds from senescing needles as accelerated senescence by heat stress has been observed previously [70]. All other tissues, in which growth during the experimental period should be assumed (e.g., roots and stem tissues), were, however, not affected by the treatment and only slight changes in C allocation between current-year needles and fine roots, and between fine roots and coarse roots were observed (Figure S1B).

At the same time, plant δ^13^C, as a marker for intrinsic water use efficiency and the relationship between A and g_s_, was largely unaffected (Figure 1). Even though our treatment clearly exceeded the optimum temperature for photosynthesis for Douglas-fir (≤20°C) [44] and we additionally induced atmospheric drought, neither the time-integrated relationship between A and g_s_ nor root and shoot biomass production were negatively affected during the treatment period. However, long-term effects of heat waves on growth remain to be investigated. Based on our observations over a 92-days treatment period, we reject our hypothesis (2) as our results point to the fact that the two provenances from the *menziesii-glauca* transition zone in southern interior British Columbia and the *glauca* zone demonstrate the physiological plasticity that allows the seedlings to continue assimilation and growth during extended periods of high temperatures in the late growing season.

### The more negative apparent respiratory fractionation indicates switches in the substrate commitment from the TCA cycle to other metabolic pathways

We did not observe increased dark respiration rates under high temperature and atmospheric drought, which supports results from other studies, such as the work by [71], who found a strong thermal acclimation of respiration in conifers but not in broad-leaved species. In the control treatment, the isotopic enrichment of respired CO_2_ above WSOM (as the putative substrate) was 3.9‰ and 5.2‰ in the Douglas-fir provenances PO and MC, respectively, and thus lies in the range observed in many other species (see review [20] and citations therein).

A decrease in the contribution of CO_2_ decarboxylated in the TCA cycle relative to CO_2_ released by PDH to total CO_2_ production will cause an increase in δ^13^C of respired CO_2_ [23] and thus a decrease in aΔ_R_. Our results (Figure 2) thus point to the fact that from a given glucose moiety entering the glycolysis/TCA cycle relatively less CO_2_ is evolved from the two TCA enzymes isocitrate dehydrogenase and 2-oxoglutarate dehydrogenase in the high temperature treatment compared to the control. Thus, there were shifts in metabolic pathways in both provenances tested toward the production of secondary metabolites (and acetogenic lipids) derived from acetyl-CoA at the expense of a more complete oxidation of the initial glucose molecule in the TCA cycle.

### High temperature induces a switch in stress protecting compounds from proline to polyols

Priault et al. [53] hypothesised that differences in the apparent respiratory fractionation between different plant functional groups can be attributed to the relative quantity of carbon committed to the TCA cycle *vs*. to secondary metabolites and acetogenic fatty acids. These authors showed that Mediterranean species producing larger amounts of secondary metabolites showed more negative apparent respiratory fractionation as compared to species investing less in these compounds. In fact, our metabolite profiling shows that the concentrations of secondary compounds and partially unsaturated fatty acids (hexadecanoic (palmitic), octadecanoic (stearic) acid) tended to increase under high temperature and VPD at the expense of TCA intermediates. The relative changes in metabolite abundance of control and stressed plants together with the assessment of apparent respiratory fractionation provide general evidence for altered metabolic priorities in both provenances under high temperature and atmospheric drought.

In general, the most abundant metabolites in all analysed tissues were sucrose, D-pinitol and quinic acid which is consistent with previous assessments of *Picea abies* [35], *Pinus nigra* and *Abies alba* [72]. Although the sucrose level was not affected by the treatment, both pinitol and quinic acid clearly increased. Polyols such as pinitol and ononitol are involved in stress responses, such as drought and heat stress, and have also been associated with osmotic adjustment [73], [74], scavenging of reactive oxygen species (ROS) [75], [76], and osmoprotection [33], [34]. [77] showed that among proline, betaine, sorbitol and mannitol, the polyols were most effective at increasing the stability of enzymes at high temperatures.

We infer that the observed increase in the two polyols is a direct acclimation response of the two Douglas-fir provenances examined to the potentially adverse effects of drought and high temperatures. In line with the increase in pinitol and ononitol is the decrease in myo-inositol and raffinose. Myo-inositol is a precursor for raffinose, ononitol and pinitol. The increased priority of polyol production most likely results in a decreased concentration of the precursor and thus potentially to restricted precursor availability for raffinose synthesis.

The increase in polyol synthesis is also related to the drop in proline concentrations under high temperature as observed in our study. Generally, proline accumulation is associated to drought and salinity stress, e.g., [26], [27], and proline functions as osmolyte, antioxidant, energy sinks and signalling molecule [78], [79]. Under these premises, we might expect proline to increase under high temperature and VPD. It has, however, also been observed that proline can be toxic to cells [28]–[31], and increased proline toxicity under heat was reported by [32]. Furthermore, proline showed to be less efficient in osmoprotection at high temperatures [77], and in ROS scavenging [80] compared to polyols. We therefore conclude that there is a preference for the trees to produce pinitol and ononitol as ROS-scavengers and osmoprotectants at the expense of proline synthesis under high temperature. In addition, the increase in alanine in N09 needles, which also may serve as osmolyte [78], might be seen in a similar light.

The concentration of monosaccharides in all analysed needle age classes (N10 and N09) increased under high temperature. Accumulation of monosaccharides was also observed in *Pinus* and *Larix* under drought [81] and in *Arabidopsis* under the combination of drought and elevated temperature [32] and after heat shock [19]. Similar to our results, Riikonen et al. [35] found an increase of monosaccharides at the expense of trisaccharides in Norway spruce seedlings exposed to slightly elevated temperature. They concluded that the increase of monosaccharides results from the remobilization of stored resources to support growth at elevated temperature. However, monosaccharide accumulation might also reflect their role in a heat stress specific replacement for proline as an osmoprotectant as previously suggested by Rizhsky et al. [32] for *Arabidopsis*.

In our study, a large portion of identified metabolites in all treatments and provenances are related to the shikimate and phenylpropanoid pathways and the treatment effect across both provenances showed a significant increase in the total amount of acids involved in the shikimic acid pathway. We observed a general trend for increases in shikimic, quinic and galactonic acid in the high temperature treatment in both provenances. Elevated levels of quinic and shikimic acid were also reported in *Arabidopsis* in response to heat [19]. In contrast to our results, a decrease in phenolics at elevated temperature was detected in Douglas-fir [35] and in other tree species [82], [83]. Quinic acid, related to phenylpropanoid synthesis, plant defence [84], [85], drought stress, and antioxidant activity [86], [87], was one of the three most abundant metabolites in our study and increased significantly in current-year needles of the provenance MC grown under high temperature. This highlights an additional facet of the heat induced metabolic responses of Douglas-fir.

In conclusion, our results strongly support our hypothesis (1), indicating that high temperature and atmospheric drought alter the preference for specific metabolic pathways and the metabolite profiles. An array of metabolites protecting against high temperature, osmotic stress, and reactive oxygen species are accumulated as well as precursors of the secondary metabolism. Such accumulation seems to occur at the expense of proline. We might interpret the metabolic acclimation as successful means to maintain physiological activity and functioning of Douglas-fir seedlings of the two examined provenances during heat waves. Concerning hypothesis (3), we can clearly state that there are no differences in biomass accumulation of seedlings from both provenances (Monte Creek and Pend Oreille). Even though the effects of increased temperature combined with atmospheric drought on the metabolism might have been slightly different between the two provenances, a general parallel trend could be observed. Apparently, the metabolic mechanisms involved in mediating resistance to elevated temperature and atmospheric drought are similar in the two provenances tested here, and we can thus reject hypothesis (3). The two provenances originated from rather dry interior areas of origin (Table 1) and we might assume that coastal provenances adapted to a more humid climate respond differently and are more sensitive to heat and drought. However, Jansen et al. [15] observed no relation between drought sensitivity and the environmental conditions at the site of origin for old-growth coastal Douglas-fir. They concluded that long-term climatic conditions at the origin averaged over the year or the growing season are poor indicators for the occurrence of stress and that the frequency of heat and drought events in a region might be better predictors for adaptation. Based on the results of the two provenances presented here, we might extend this hypothesis to seedlings. In conclusion, we can state that the Douglas-fir seedlings from the *glauc*a zone and *menziesii*-*glauca* transition assessed here are well adapted to extended periods of high air temperature and atmospheric drought and thus might represent a suitable resource for forestry in Central Europe under future climatic conditions and extreme events.

## Supporting Information

Figure S1 **Effects of increased temperature on Douglas-fir provenances total and organ-specific biomass (A) and on biomass ratios between organs (B).** (A) shows absolute dry weight data of seedlings of the provenances Pend Oreille and Monte Creek grown under control conditions (C; 20°C, 85% rH) and elevated temperature and increased VPD (T; 30°C, 55% rH). Data shown are mean values ± SD (N = 4 to 5). Small letters indicate homogeneous groups (Tukey posthoc test). Large letters indicate significant effects of provenance (P) and treatment (T) (ANOVA). FR, fine roots, CR, coarse roots, B, bark, W, wood, TW, twig, N10, current year needles (2010), N09 and N08, previous year needles (2009, 2008, respectively).
(TIF)

Table S1 **Growing conditions during the acclimatization and treatment phase in the walk-in climate chambers with the control (C) and the treatment (T) conditions.**
(DOCX)
