# Supplementary figures and images for: Douglas-Fir Seedlings Exhibit Metabolic Responses to Increased Temperature and Atmospheric Drought
Source: PLoS One. 2014 Dec 1;9(12):e114165. doi: 10.1371/journal.pone.0114165 (PMC4250086; doi:10.1371/journal.pone.0114165)

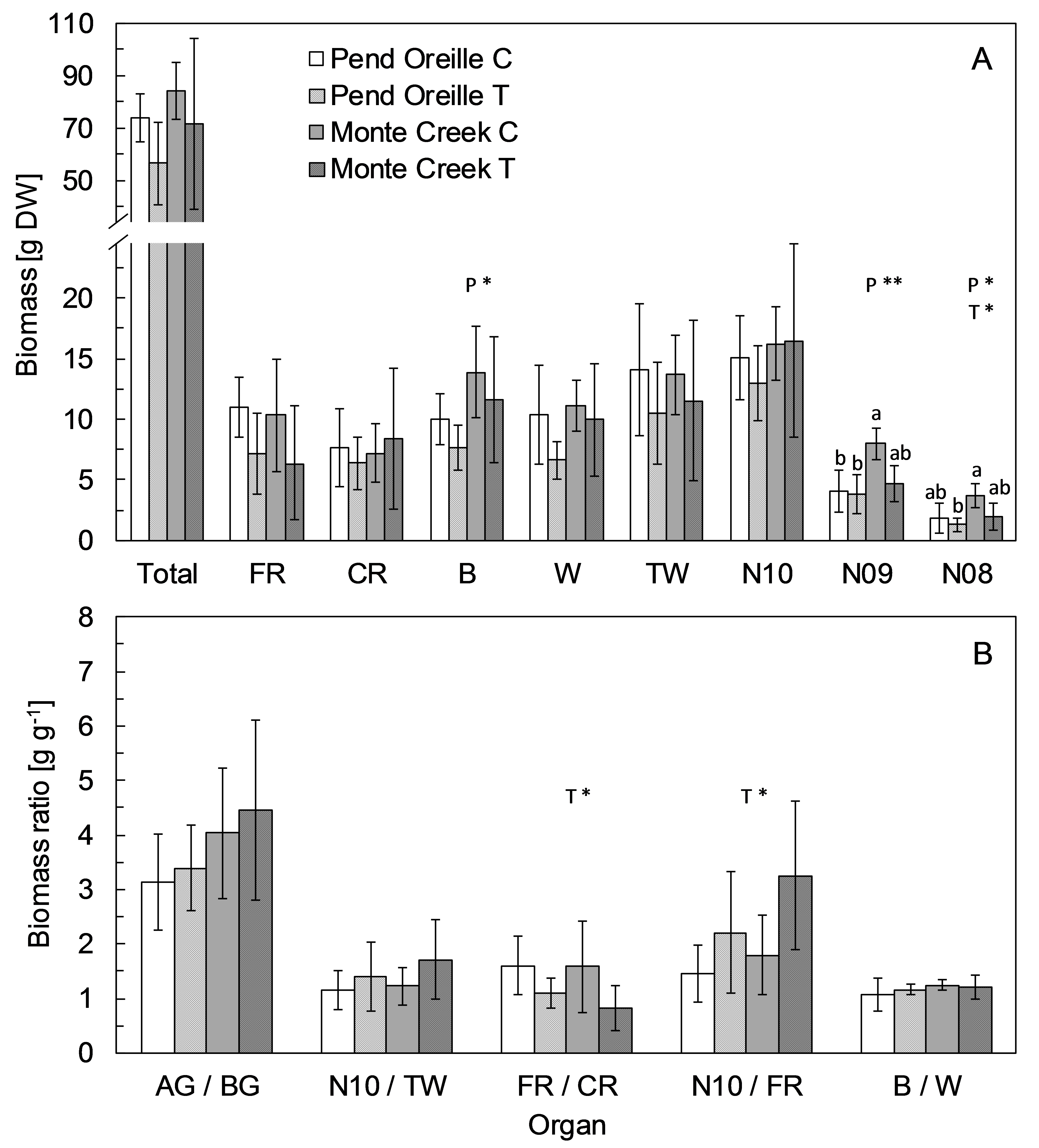

Supplement: Figure S1 — Effects of increased temperature on Douglas-fir provenances total and organ-specific biomass (A) and on biomass ratios between organs (B). (A) shows absolute dry weight data of seedlings of the provenances Pend Oreille and Monte Creek grown under control conditions (C; 20°C, 85% rH) and elevated temperature and increased VPD (T; 30°C, 55% rH). Data shown are mean values ± SD (N = 4 to 5). Small letters indicate homogeneous groups (Tukey posthoc test). Large letters indicate significant effects of provenance (P) and treatment (T) (ANOVA). FR, fine roots, CR, coarse roots, B, bark, W, wood, TW, twig, N10, current year needles (2010), N09 and N08, previous year needles (2009, 2008, respectively). (TIF) [file pone.0114165.s001.tif]
