# Supplementary material for: Douglas-Fir Seedlings Exhibit Metabolic Responses to Increased Temperature and Atmospheric Drought
Source: PLoS One. 2014 Dec 1;9(12):e114165. doi: 10.1371/journal.pone.0114165 (PMC4250086; doi:10.1371/journal.pone.0114165)
Supplement: Table S1 — Growing conditions during the acclimatization and treatment phase in the walk-in climate chambers with the control (C) and the treatment (T) conditions. (DOCX) [file pone.0114165.s002.docx]

**SUPPLEMENTARY MATERIAL**

Table S1. Growing conditions during the acclimatization and treatment phase in the walk-in climate chambers with the control (C) and the treatment (T) conditions

|  | **Acclimatization phase** | | | | **Treatment phase** |
| --- | --- | --- | --- | --- | --- |
|  | **[d before treatment start]** | | | | **[d after treatment start]** |
|  | **-78** | **-72** | **-68** | **-3** | **0-92** |
| Photosynthetic photon flux density [µmol m^-2^ s^-1^] | 250 ±50 | 250 ±50 | 250 ±50 | 400 ±50 | 600 ±50 |
| Photoperiod [h day^-1^] | 12 | 14 | 16 | 16 | 16 |
| Air temperature [°C day/night] | 10/10 | 15/13 | 20/15 | 20/15 (C) | 20/15 (C) |
|  |  |  |  | 25/20 (T) | 30/25 (T) |
| Relative air humidity [% day] | 85 ±5 | 85 ±5 | 85 ±5 | 85 ±5 (C) | 85 ±5 (C) |
|  |  |  |  | 65 ±5 (T) | 55 ±5 (T) |
| Air vapour pressure deficit [kPa] |  |  |  |  | 0.35 ±0.1 (C) |
|  |  |  |  |  | 1.91 ±0.2 (T) |
